# The slan antigen identifies the prototypical non-classical CD16^+^-monocytes in human blood

**DOI:** 10.3389/fimmu.2023.1287656

**Published:** 2023-10-27

**Authors:** Nicola Tamassia, Francisco Bianchetto-Aguilera, Sara Gasperini, Alessio Grimaldi, Claudia Montaldo, Federica Calzetti, Elisa Gardiman, Ilaria Signoretto, Monica Castellucci, Vincenzo Barnaba, Marco Tripodi, Marco Antonio Cassatella

**Affiliations:** ^1^ Department of Medicine, Section of General Pathology, University of Verona, Verona, Italy; ^2^ Department of Internal Clinical Sciences, Anesthesiology and Cardiovascular Sciences, Sapienza University of Rome, Rome, Italy; ^3^ Department of Molecular Medicine, Sapienza University of Rome, Rome, Italy; ^4^ National Institute for Infectious Diseases L. Spallanzani, IRCCS, Rome, Italy; ^5^ Centro Piattaforme Tecnologiche, University of Verona, Verona, Italy

**Keywords:** slan^+^-monocytes, classical/non-classical monocytes, transcriptomics, scRNA-seq, proteomics

## Abstract

**Introduction:**

Peripheral monocytes in humans are conventionally divided into classical (CL, CD14^++^CD16^−^), intermediate (INT, CD14^++^CD16^+^) and non-classical (NC, CD14^dim/−^CD16^++^) cells, based on their expression levels of CD14 and CD16. A major fraction of the NC-monocytes has been shown to express the 6-sulfo LacNAc (slan) antigen, but whether these slan^+^/NC-monocytes represent the prototypical non-classical monocytes or whether they are simply a sub-fraction with identical features as the remainder of NC monocytes is still unclear.

**Methods:**

We analyzed transcriptome (by bulk and single cell RNA-seq), proteome, cell surface markers and production of discrete cytokines by peripheral slan^+^/NC- and slan^−^/NC-monocytes, in comparison to total NC-, CL- and INT- monocytes.

**Results:**

By bulk RNA-seq and proteomic analysis, we found that slan^+^/NC-monocytes express higher levels of genes and proteins specific of NC-monocytes than slan^−^/NC-monocytes do. Unsupervised clustering of scRNA-seq data generated one cluster of NC- and one of INT-monocytes, where all slan^+^/NC-monocytes were allocated to the NC-monocyte cluster, while slan^−^/NC-monocytes were found, in part (13.4%), within the INT-monocyte cluster. In addition, total NC- and slan^−^/NC-monocytes, but not slan^+^/NC-monocytes, were found by both bulk RNA-seq and scRNA-seq to contain a small percentage of natural killer cells.

**Conclusion:**

In addition to comparatively characterize total NC-, slan^−^/NC- and slan^+^/NC-monocyte transcriptomes and proteomes, our data prove that slan^+^/NC-, but not slan^−^/NC-, monocytes are more representative of prototypical NC-monocytes.

## Introduction

Human peripheral monocytes from healthy donors (HDs) are currently divided into three subgroups, namely the “classical” (CL) CD14^++^CD16^−^ monocytes (accounting for approximately 85% of the total monocytes), the “non-classical” (NC) CD14^dim/−^CD16^++^ monocytes (approximately 10%) and the “intermediate” (INT) CD14^++^CD16^+^monocytes (approximately 5%) (1). Such a subgroup division relies on flow cytometry gating strategies that, however, not always are employed by researchers according to unanimously accepted criteria. In other words, NC-monocytes might not be appropriately gated, and this may result in contrasting data reported by the literature about their frequencies in diseases (2). Another complication affecting the detection and, in turn, the correct evaluation of monocyte subgroups is that CD14 and CD16 expression may change upon monocyte interaction with various endogenous/exogenous stimuli (2, 3). Therefore, more stable markers are required to more accurately determine either the frequency and/or the eventual alteration of monocyte subgroups under pathological conditions.

This said, 50/60% of the NC-monocytes typically express the 6-Sulfo LacNAc (slan) antigen (3, 4), which is an O-linked glycosylated variant of P-selectin glycoprotein ligand-1 (PSGL-1) recognized by specific monoclonal antibodies, including MDC8, DD1 and DD2 (4–6). These NC-monocytes are currently known as slan^+^-monocytes (3), but have been for long referred to as slan^+^-dendritic cells (slanDCs) (6) until molecular studies demonstrated their typical monocyte, but not DC, transcriptome (7–9). In a recent review, the specific literature on NC- and slan^+^/NC-monocytes has been side-by-side analyzed in terms of phenotypical and functional features, as well as behavior in human diseases, ultimately concluding that no substantial differences exist between them (2). Therefore, the slan marker has been recently proposed for definition of NC-monocytes (as CD16^+^slan^+^-cells) (2). However, NC-monocytes also contain 40 to 50% of CD14^dim^CD16^+^slan^−^-monocytes (3, 10, 11) not included in INT-monocytes, whose molecular, phenotypical and functional relationship to either total NC-monocytes or slan^+^/NC-monocytes, is poorly established. In such regard, by performing bulk transcriptomic studies, Cros et al. (12) concluded that slan^+^/NC- and slan^−^/NC- monocytes cannot be discriminated between themselves. By contrast, a more recent RNA-seq study described 20 upregulated genes in slan^+^/NC-monocytes compared to slan^−^/NC-monocytes (11).

Herein, by performing bulk and single-cell RNA-seq, we confirm a substantial similarity between slan^−^/NC- and slan^+^/NC-monocytes, but also uncover some distinguishing molecular features between them. Furthermore, by proteomic studies, we show that, in comparison to slan^+^/NC-monocytes, slan^−^/NC-monocytes express higher levels of discrete proteins that are also present in CL-monocytes, including ELANE, MPO and S100A9. Altogether, our data indicate that slan^+^/NC-monocytes, being the cell population displaying the greatest transcriptomic and proteomic differences with the CL-monocytes, represent prototypical NC-monocytes.

## Materials and methods

### Cell isolation

Peripheral blood mononuclear cells (PBMCs) were isolated from buffy coats of HDs by density centrifugation over Ficoll-Paque gradient (GE Healthcare, Little Chalfont, Buckinghamshire, United Kingdom), under endotoxin-free conditions (8). Thereafter, monocyte subgroups were isolated from PBMCs by fluorescence activated cell sorting [using a FACS ARIA FUSION flow cytometer (Becton Dickinson, Franklin Lakes, NJ)] at a very high purity (> 97%), using the following antibodies: anti-CD14 VioBlue (REA599, Miltenyi Biotec, Bergisch Gladbach, Germany), anti-CD16 PerCP-Cy5.5 (3G8, BioLegend, San Diego, CA, USA), anti-HLA-DR APC-Cy7 (L243, Biolegend), anti-M-DC8 FITC (DD1, Miltenyi Biotec), anti-CD45 Brilliant Violet 510 (HI30, BioLegend), as well as, as lineage exclusion markers, anti-CD56 PE (REA196, Miltenyi Biotec), anti-CD3 PE-Vio770 (REA613, Miltenyi Biotec) and anti-CD19 PE-Vio 770 (REA675, Miltenyi Biotec). The gating strategy used to isolate monocyte subgroups is shown in **Supplementary Figure 1**.

### RNA sequencing (RNA-seq)

Total RNA was extracted by the RNeasy Mini Kit (Qiagen) after cell lysis (13). Libraries for transcriptome analysis were prepared using the Smart-seq2 protocol (14), as recently described (15). Libraries were sequenced by the Illumina NextSeq 500 in single-read mode (1x75 cycles) at Centro Piattaforme Tecnologiche (CPT) of the Verona University.

### Single-cell RNA sequencing (scRNA-seq)

Sorted monocyte subgroups (CL-, INT-, total NC-, slan^−^/NC- and slan^+^/NC-monocytes) were labeled by using the BD Single-Cell Multiplexing Kit (BD Biosciences), strictly following the manufacturer’s protocol (BD Biosciences). Then, each of the monocyte subgroups was washed twice in FACS buffer (PBS with 2% FBS and 2 mM EDTA) and then resuspended in cold BD Sample Buffer (BD Biosciences). Only samples with more than 85% viable cells were used for sequencing. Monocyte subgroups from the same donor were then pooled to achieve approximately 15000 cells in 620 µl, and then loaded onto a BD Rhapsody cartridge for an incubation of 20 min at room T. Then, Cell Capture Beads (BD Biosciences) were added to the cartridge, incubated at room T for 3 min, and thereafter cartridges were washed. Cells were then lysed, and the released mRNAs captured by Cell Capture Beads. mRNAs were then retrieved, to be washed prior to performing reverse transcription and treatment with Exonuclease I. cDNA Libraries were prepared by using the BD Rhapsody Whole Transcriptome Analysis (WTA), Amplification kit and BD Single-Cell Multiplexing kit (BD Biosciences). Quality of final libraries was assessed by Agilent 2200 TapeStation with High Sensitivity D5000 ScreenTape and quantified using a Qubit Fluorometer using the Qubit dsDNA HS Kit (Thermo Fisher, #Q32854). Sequencing was performed in paired-end mode (2x75 cycles) on a NextSeq 500 System (Illumina). This procedure was utilized for CL-, INT-, total NC-, slan^−^/NC- and slan^+^/NC-monocytes isolated from two HDs and the resulting data were integrated as outlined below.

### RNA-seq computational analysis

Computational analysis of transcriptome datasets generated by Smart-seq2 has been performed by using the bioinformatic pipeline, as previously described (8). Briefly, after quality filtering, according to the Illumina pipeline, removal of contaminant adapters and base quality trimming were performed using Trim Galore! (http://www.bioinformatics.babraham.ac.uk/projects/trim_galore/) script with the length parameter set to 50. Trimmed reads were quantified using Kallisto quant (16) applying parameters -bias -single -l 200 -s 20. Transcript quantification obtained from Kallisto was combined to gene level using tximport packages v1.22.0. Gene counts were normalized among various samples using DESeq2 (17) v1.26.0, and only genes coding for protein and long non-coding RNA (lncRNA) were retained for downstream analysis. Differentially expressed genes (DEGs) were identified using DESeq2, by using as selection parameter an adjusted *P* value lower than 0.01 and likelihood ratio test (LRT) (17). Batch effects were removed using the limma v3.42.2 package’s “removeBatchEffect” function before performing principal component analysis (PCA). PCA was performed on DEGs by using Bioconductor/R package pcaExplorer v.2.12.0 and hierarchical clustering was performed by R package stats v3.6.3 using the Euclidean distance and complete aggregation as criteria.

### Seven bridges processing for scRNA-seq data

After demultiplexing of bcl files by using Bcl2fastq2 V2.20 from Illumina and assessment of reads quality, paired-end scRNA-seq reads were then filtered for valid cell barcodes using the barcode whitelist provided by BD. Then, sequenced reads were aligned to the hg38 human transcriptome and the expression of transcripts in each cell was quantified *via* the standard Rhapsody analysis pipeline (BD Biosciences) on Seven Bridges (https://www.sevenbridges.com), following manufacturer’s recommendations.

### Seurat workflow for scRNA-seq data analysis

The R package Seurat v3.2.2 was utilized for all downstream analysis of scRNA-seq data. For each single cell dataset, the number of detected genes, the number of unique molecular identifiers (UMIs), as well as the fraction of UMIs corresponding to mitochondrial features, which altogether reflect the transcriptome quality of each cell, were calculated. Only cells that transcribed at least 200 genes, and only genes that were expressed in at least 10 cells, were included in the analysis. In sum, 20890 cells and 13574 genes were obtained. Then, after quality control, a total of 19085 cells were analyzed (8691 from donor 1 and 10394 from donor 2). To remove batch effects across data from different donors, we performed dataset integration using the standard Seurat integration workflow (https://satijalab.org/seurat/archive/v3.2/integration.html). To identify the integration of anchor genes among the 2 datasets from different donors, the FindIntegrationAnchors() function was used by applying default parameters. Using Seurat’s IntegrateData(), samples were combined into one object. These ‘integrated’ batch-corrected values were then set as the ‘default assay’, and gene expression values were scaled before running PCA. The dimensional reduction of the integrated dataset was computed by summarizing the first 20 PCs and visualized in a two-dimensional UMAP representation. Clustering was conducted using the FindNeighbors() and FindClusters() functions using the same 20 PCs, and a resolution parameter set to 0.3. Differential expression (DE) tests were performed using FindAllMarkers() function. DEGs were identified using the non-parametrical Wilcoxon Rank Sum test, based on normalized data. *P* value adjustment was performed using Bonferroni correction based on the total number of genes in the dataset. Genes with > 0.25 log-fold changes, expressed in at least 25% of the cells in the tested groups, and Bonferroni-corrected *P* values < 0.01 were considered as significant DEGs. The average gene expression of clusters was calculated using the function AverageExpression().

The non-classical monocyte space was investigated by subsetting the scRNA-seq dataset to that cluster identified as c6 and repeating the integration step of 2 datasets from different donors, regression for the percentage mitochondrial genes and scaling as described above. The dimensionality of the data was then reduced to 30 PCs, which was served as input for the UMAP calculation. The SNN-graph Louvain clustering of non-classical monocytes was performed using a resolution of 0.35. The Differential Expression test was performed using the same criteria described above.

### Cytokine detection

Cytokine concentrations in cell-free supernatants were measured by a customized Human Luminex Discovery Assay (R&D Systems, Minneapolis, MN, USA), specific for human IL-12, TNF, IL-6, CCL2, CXCL10 and CCL20. Acquisition of fluorescence emissions was performed by using Luminex instruments and analyzed with Bio-plex manager (Bio-Rad) software.

### Protein digestion, peptide purification and nanoLC analysis

Cell pellets from CL-, total NC-, slan^−^/NC- and slan^+^/NC-monocytes (1 x 10^6^ cells/subset) were lysed in 20 µL of 0.5% NP-40, 10% Glycerol, 150 mM NaCl and 10 mM Tris-HCl pH 8 supplemented with cOmplete™ Protease Inhibitor Cocktail (Roche), 1 mM Na_3_VO_4_, 5 mM NaF and 1 mM PMSF. Protein lysates were then treated with 10 mM DL-Dithiothreitol and incubated for 30 min at 56° for cysteine reduction, while the cysteines were alkylated with 55 mM Iodoacetamide (20 min at RT, in the dark). Samples were precipitated overnight at +4° by using five volumes of 100% ethanol and resuspended in 40 μl of 50 mM ammonium bicarbonate and 2 M Urea. Protein mixture was digested overnight at 37° with 1.5 μl of 0.2 μg/20 µL of trypsin solution. Peptide mixtures were desalted and filtered through a C18 microcolumn ZipTip, and then eluted from the C18 bed using 10 μL of 80% ACN/0.1% TFA. Organic component was again removed by evaporating in a vacuum centrifuge and peptides resuspended in a suitable nanoLC injection volume of 2.5% ACN/0.1% TFA and 0.1% formic acid. After 5 min of sonication, peptides were analyzed by an Ultimate 3000 RSLCnano system (Dionex, Sunnyvale, CA, USA) equipped with a splitting cartridge for nanoflow and connected on-line *via* a nano-ESI source to an Q Exactive plus™ Hybrid Quadrupole-Orbitrap™ Mass Spectrometer (Thermo-Fisher Scientific). 17 μl of each peptide mixture were automatically loaded onto a pre-column cartridge for peptide concentration. Peptides were separated on a 15 cm long analytical easy-spray column (PepMap^®^ RSLC, C18, 3 μm o.d., 100 Å, 75 μm X150mm, Thermo Fisher Scientific). The mobile phase A was 0.1% formic acid and mobile phase B was 0.1% formic acid in 80% acetonitrile. The multistep elution gradient was the following: from 4% to 25% of phase B within 55 min, from 25% to 40% of B in 15 min, from 40% to 90% of B in 5 min at a constant flow rate of 300 nl/min. Eluted peptides were electrosprayed directly into the mass spectrometer with an ESI voltage of 2.0 kV. MS data were acquired in a positive mode in the Orbitrap in FTMS mode over 350-1700 *m/z* range with resolution 70,000, with an automatic gain control (AGC) target of 3×10^6^ ions, and the maximal injection time of 100 ms. Tandem mass spectra were acquired into the linear ion trap quadrupole (ITMS) by data-dependent mode with the Excalibur software (Thermo-Fisher Scientific), selecting the fifteen most intense ions with charge states 2,3,4,5 and 6, through collision-induced dissociation (CID), and analyzing the resulting fragments in the Orbitrap mass analyzer. For MS/MS scanning, target value to 10000 ions and injection time of 80 ms. All MS/MS spectra were collected using a normalized collision energy of 30% and an isolation window of 2 m/z. To avoid redundant sequencing of the most abundant peptides, a dynamic exclusion duration of 20 seconds was selected. Proteins were automatically identified using the proteomics software package MaxQuant (version 1.6.17.0). Tandem mass spectra were searched against the *Homo sapiens* dataset of UniprotKB database (Release: Feb 2016; 550,552 sequences). Trypsin was selected as cleavage enzyme. A maximum of 3 missed cleavages was allowed. Mass tolerance for FTMS and for ITMS measurements were respectively set to 20 ppm and 0.5 Da. The False Discovery Rate (FDR) for proteins and peptides identification was set to 1%. Carbamidomethylation of cysteine was set as a fixed modification. The following variable modifications were used for both identification and quantification: oxidation of methionine; lysine acetylation. Minimum peptides length was set to 7 amino acids. For protein quantification was enabled the label free quantification (LFQ) algorithm. Searches were also implemented querying dataset of commonly detected contaminants in proteomics as well as the reverse decoy database generated by the Andromeda search engine. The comparisons among CL-, total NC-, slan^−^/NC- and slan^+^/NC-monocytes were performed using the LFQ protein intensities calculated by MaxQuant (http://www.maxquant.org). Statistical analyses for proteomic data were performed by using freely available Perseus software (version 1.6.7.0) after log2 transformation of the intensity data. Each sample group was analyzed in replicate. Statistical analysis was carried out on proteins identified in 100% of the samples. In sample t test and volcano plot analysis a *P* value ≤ 0.05 was accepted as significant.

### Flow cytometry intracellular staining

0.5x10^6^ PBMCs were suspended in 100 µl of PBS buffer (Corning) plus 2% FBS and 2mM EDTA (Sigma-Aldrich) (from now on termed ‘staining buffer’) and subsequently incubated for 10 minutes in the presence of 5% human serum (Sigma-Aldrich). Firstly, for surface antigens detection, cells were stained with anti-CD14 VioBlue (REA599, Miltenyi Biotec), anti-CD45 Brillant Violet 510 (HI30, BioLegend), anti-MDC8 FITC (DD-1, Miltenyi), anti-CD56 PE-Vio 615 (REA196, Miltenyi Biotec), anti-CD16 PerCp-Cy5.5 (3G8, BioLegend), anti-CD3 PE-Vio 770 (REA613, Miltenyi Biotec), anti-CD19 PE-Vio 770 (REA675, Miltenyi Biotec) and HLA-DR APC-Cy7 (L243, Biolegend) for 30 min on ice. Then, PBMCs were fixed and permeabilized by eBioscience Foxp3/Transcription Factor Staining Buffer Set according to the manufacturer’s instructions (Thermo Fisher Scientific). Subsequently, antibody unspecific staining was blocked by 2% human serum and finally cells were stained for intracellular antigens using the following antibodies anti-MRP-14 (S100A9) APC (MRP 1H9, BioLegend), anti-hNeutrophil Elastase Alexa Fluor 647 (R&D Systems), anti-MPO APC (REA491, Miltenyi Biotec) for 30 min at RT. Sample fluorescence was then measured by a 14-color MACSQuant16 Analyzer (Miltenyi Biotec) flow cytometer, while data analysis was performed using FlowJo software version 10 from Tree Star (Ashland, OR, USA).

### Statistical analysis

Data are expressed as means ± SEM. Statistical evaluation was performed using one-way ANOVA or two-way ANOVA followed by Tukey’s *post hoc* test or Holm-Sidak’s multiple comparisons test, respectively. Values of *P* < 0.05 were considered statistically significant. Statistical analysis was performed by GraphPad Prism Version 9 software (GraphPad Software, Inc.).

### Data availability

The RNA-seq and scRNA-seq datasets generated in this study are available at the Gene Expression Omnibus database (https://www.ncbi.nlm.nih.gov/geo/) under the accession numbers GSE136107 and GSE241266.

## Results

### Analysis of discrete functional responses by NC-, slan^−^/NC- and slan^+^/NC-monocytes

**Figure 1:**
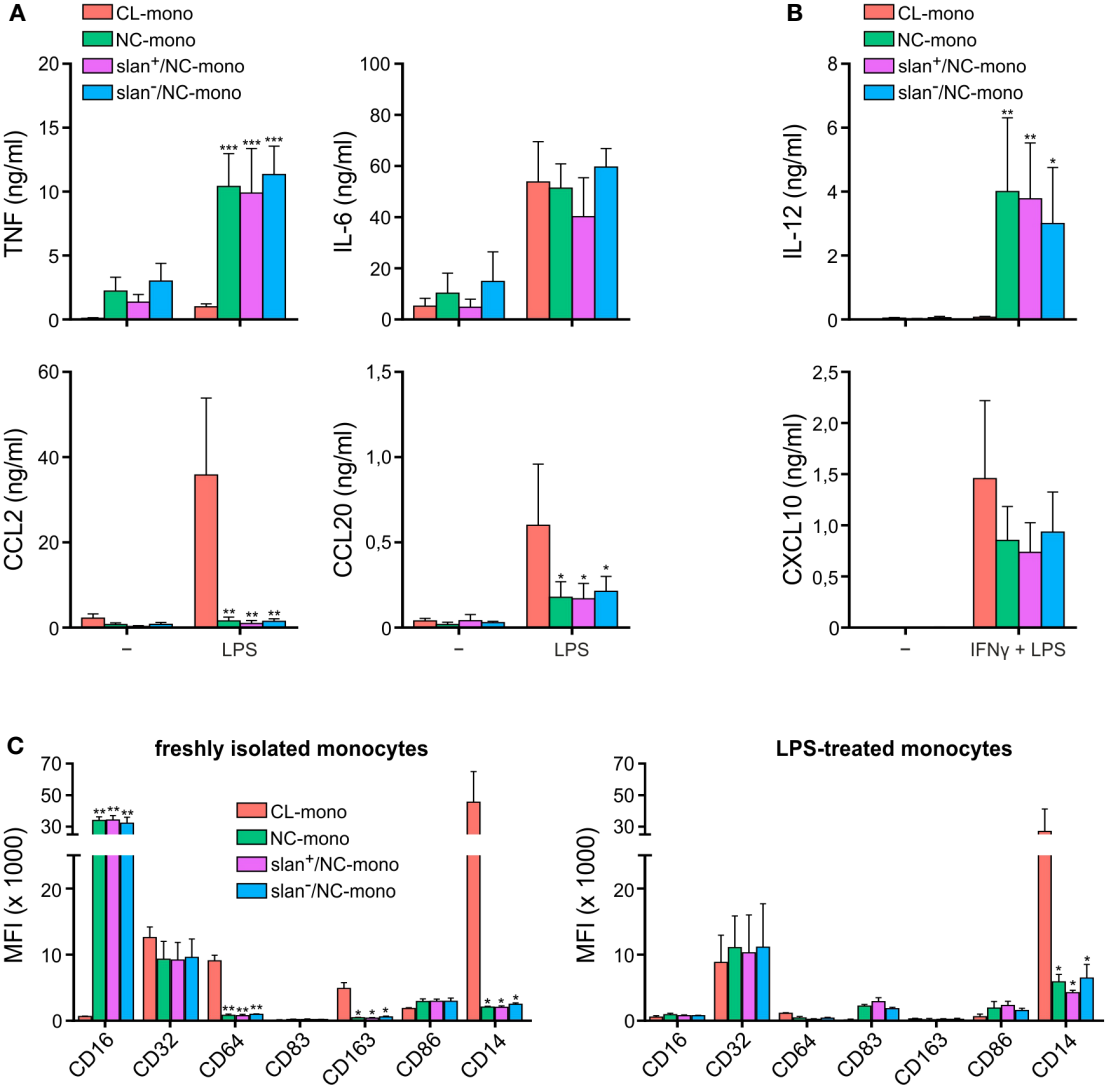
Cytokine production and antigen expression by CL-, total NC-, slan^−^/NC- and slan^+^/NC-monocytes. Production of TNF, IL-6, CCL2 and CCL20 **(A)**, or IL-12 and CXCL10 **(B)**, by CL-, total NC-, slan^−^/NC- and slan^+^/NC-monocytes, sorted as depicted in **Supplementary Figure 1** and then incubated with either 100 ng/ml LPS for 20 h **(A)**, or pretreated with 200 U/ml IFNγ for 15 h and then incubated with 100 ng/ml LPS for the subsequent 20 h **(B)**. Supernatants were collected, and cytokine/chemokine levels measured by Luminex assays. Results are expressed as the mean values ± SEM (n=4). Asterisks indicate significant differences compared to CL (*P < 0.05, **P < 0.01, ***P < 0.001 by two-way ANOVA corrected for Holm-Sidak’s multiple comparisons test). **(C)** CL-, total NC-, slan^−^/NC- and slan^+^/NC-monocytes either freshly isolated (left panel) or incubated with 100 ng/ml LPS for 20 h (right panel) were stained by specific fluorochrome-conjugated antibodies to evaluate CD16, CD32, CD64, CD83, CD163, CD86, and CD14 membrane expression by flow cytometry. Bar plots show the median of the mean fluorescence intensity (MFI) ± SEM (n = 3). Asterisks indicate significant differences compared to CL (*P < 0.05, **P < 0.01,***P < 0.001 by one-way ANOVA followed by Tukey’s post test).

In initial experiments, freshly isolated NC-, slan^−^/NC- and slan^+^/NC-monocytes were sorted along with CL-monocytes according to the gating strategy shown in **Supplementary Figure 1**, to compare their cytokine production pattern upon stimulation. As shown in **Figure 1**, custom-made multiplex cytokine assays revealed no significantly different capacity among total NC-, slan^−^/NC- and slan^+^/NC-monocytes to produce either TNF, IL-6, CCL2 and CCL20 in response to a treatment for 20 h with LPS (**Figure 1A**), or IL-12 and CXCL10 in response to IFNγ plus LPS (**Figure 1B**). On the other hand, these experiments confirmed (12, 18, 19) that, while NC/slan^−^/slan^+^-monocytes produce IL-12 and TNF in greater amounts than CL monocytes, the latter cells produce greater amounts of CCL2 and CCL20 (**Figures 1A, B**). NC-, slan^−^/NC- and slan^+^/NC-monocytes were also found not to significantly differ in terms of CD16, CD32, CD64, CD83, CD86, CD163 and CD14 expression, if markers were measured either right after cell isolation (**Figure 1C**, left panel), or after an incubation with LPS for 20 h (**Figure 1C**, right panel). Notably, these experiments also confirmed (20, 21) that CD16 expression dramatically decreases in NC/slan^−^/slan^+^-monocytes incubated with LPS (**Figure 1C**), once again demonstrating that CD16 cannot be always used for a correct identification and quantification of NC-monocytes. Altogether, these experiments show that NC-, slan^−^/NC- and slan^+^/NC-monocytes display a similar capacity to produce high amount of TNF, IL-6, IL-12, CXCL10, CCL2 and CCL20, as well as to display identical phenotypes after isolation from the blood or upon incubation with LPS, at least with regards to CD16, CD32, CD64, CD83, CD86, CD163 and CD14 expression.

### Transcriptomic profiles of NC-, slan^−^/NC- and slan^+^/NC-monocytes by bulk RNA-seq

**Figure 2:**
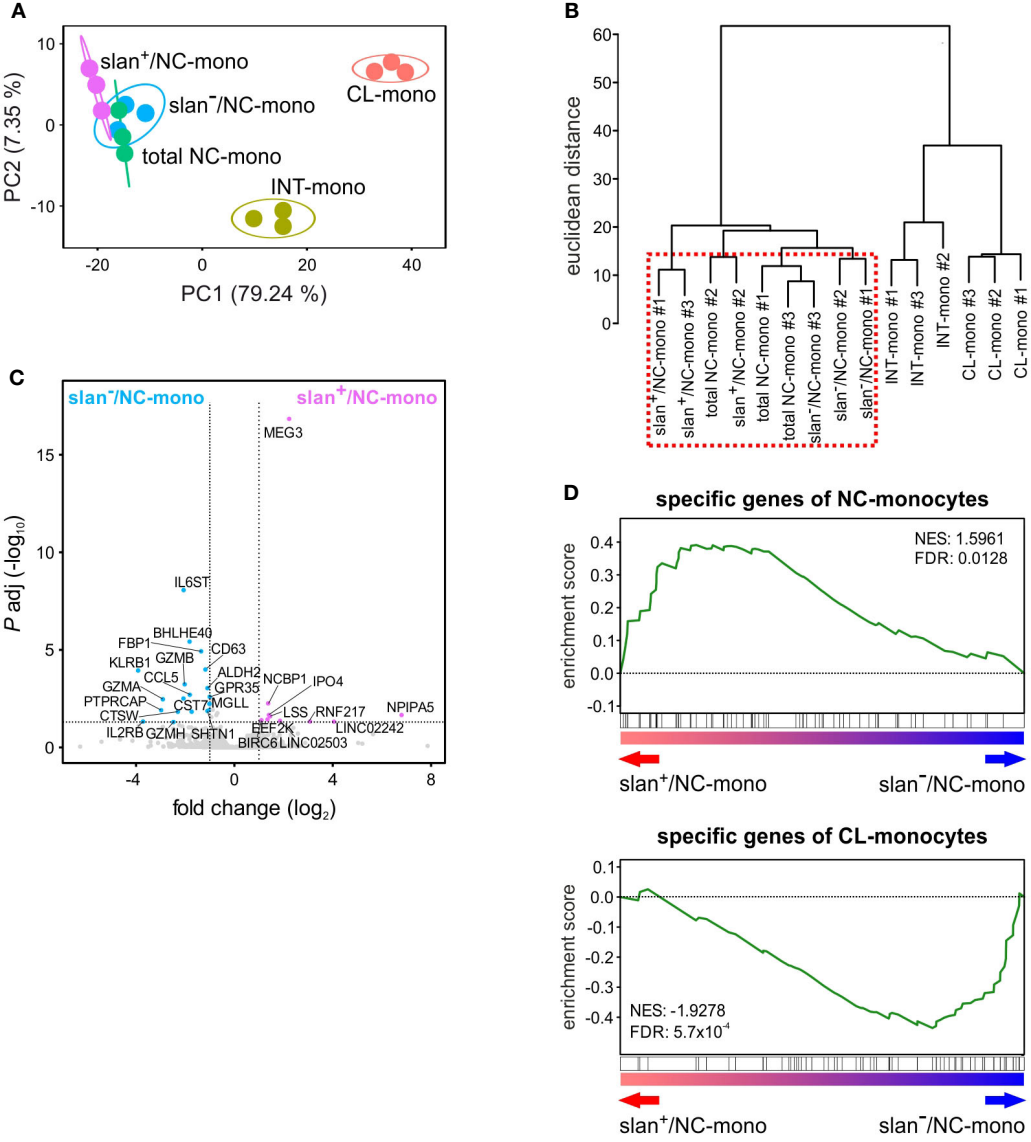
Gene expression profiles obtained by bulk RNA-seq studies of monocyte subgroups. Circulating monocyte subgroups were isolated by the gating strategy shown in **Supplementary Figure 1** and subjected to RNA-seq experiments. **(A)** PCA scatter plot based on the differentially expressed genes (DEGs) identified from bulk RNA-seq analyses of CL- (salmon), INT- (yellow ochre), total NC- (green), slan^+^/NC- (purple) and slan^−^/NC- (light blue) monocytes (n = 3). **(B)** Dendrogram of the unsupervised hierarchical clustering analysis based on DEGs identified from bulk RNA-seq analyses of monocyte subgroups. The red dashed box highlights that total NC-, slan^+^/NC- and slan^−^/NC-monocytes display similar transcriptomes. **(C)** Volcano plot displaying DEGs between slan^+^/NC- and slan^−^/NC-monocytes. DEGs more expressed (*P* < 0.01 and fold change > 2) in slan^+^/NC- and slan^-^/NC-monocytes are marked by, respectively, purple and light-blue dots, while genes not significantly different are shown as grey dots. Each dot represents the mean value of three independent experiments. **(D)** GSEA plots displaying the enrichment score in slan^+^/NC- and slan^−^/NC-monocytes of specific genes from CL- (upper plot) and NC- (lower plot) monocytes retrieved from Anbazhagan et al.’s study (22). The false discovery rate (FDR) and normalized enrichment score (NES) were also estimated for each monocyte signature.

We then sorted total NC-, slan^−^/NC- and slan^+^/NC-monocytes along with CL- and INT-monocytes for bulk RNA-seq experiments to compare their transcriptomes. By applying the likelihood ratio test (LRT), we identified 2417 DEGs (> 1 FPKM, *P*adj < 0.01) among the various monocyte subgroups. Confirming and extending previous data (8, 12), Principal Component Analysis (PCA) revealed three main groups corresponding to CL-, INT- and NC-monocytes (**Figure 2A**). Here, the total NC-, the slan^+^/NC- and slan^−^/NC-monocytes are all located to the upper left corner of the PCA plot, even though slan^+^/NC- and slan^−^/NC-monocytes are separated within this space. Moreover, hierarchical clustering confirmed that the transcriptomes of total NC-, slan^+^/NC- and slan^−^/NC-monocytes segregate together, due to a high degree of similarity (**Figure 2B**). However, Wald test analysis restricted to slan^+^/NC- and slan^−^/NC-monocytes identified 29 DEGs, 10 of them more expressed in slan^+^/NC-monocytes, the remaining 19 more expressed in slan^−^/NC-monocytes (**Figure 2C**). Among DEGs increased in slan^+^/NC-monocytes, 3 genes were long noncoding RNAs (lncRNAs), i.e., *LINC02242*, *LINC02503* and *MEG3* (**Figure 2C**), the latter showing a 4.6 fold higher expression in slan^+^/NC-monocytes. On the other hand, genes downregulated in slan^+^/NC-monocytes as compared to slan^-^/NC-monocytes included *IL6ST*, the gp130 b-subunit of the IL-6 receptor (4.2 fold), *CD63* (2.3 fold) and the *CCL5* chemokine (3.5 fold). These differences in gene expression suggest different roles of slan^+^/NC- and slan^−^/NC-monocytes in immune response. Of note, 4 of the 20 DEGs in slan^−^/NC-monocytes (i.e., *GZMA, GZMB, GZMH* and *KLRB1)* were found to represent genes more specific for NK cells than for monocytes (**Figure 2C**). This latter finding is consistent with results from other papers focused on the transcriptomes of total NC-monocytes (19, 23), and is likely explained by a potential contamination of slan^−^/NC-monocytes with the recently described CD16^+^CD56^−^-NK cells (24). In fact, when a sorting strategy utilizing anti-CD56 antibodies to exclude NK cells is used, then CD16^+^CD56^−^-NK cells can be found in total NC- and slan^−^/NC-monocytes (that are selected for CD16 expression), but not in slan^+^/NC-monocytes. This hypothesis is corroborated by our scRNA-seq experiments (see subsequent paragraphs), which identified a cluster of NK cells (i.e., c7) in total NC- and slan^−^/NC-, but not in slan^+^/NC-, monocytes.

Next, by using Gene Set Enrichment Analysis (GSEA) (25), we investigated which cell population, between slan^+^/NC- and slan^−^/NC-monocytes, accumulates higher levels of genes belonging to a NC-monocyte signature retrieved from an *ad hoc* review summarizing data from five different studies (22). As shown in **Figure 2D**, while slan^+^/NC-monocytes were found to accumulate more transcripts belonging to the NC-monocyte gene signature (top panel), slan^−^/NC-monocytes were found to accumulate more transcripts belonging to the CL-monocyte signature (lower panel).

In sum, bulk transcriptomic studies demonstrated that, even if slan^+^/NC- and slan^−^/NC-monocytes display highly similar transcriptomes, slan^+^/NC-monocytes express higher levels of the NC-monocyte signature genes than slan^−^/NC-monocytes do. Moreover, our data support the notion of more reliable isolation/sorting of NC-monocytes *via* the slan marker, since the method based on CD16 and CD14 expression seems to favor the sorting of few contaminating CD16^+^CD56^−^-NK cells.

### slan^+^/NC- and slan^−^/NC-monocytes display different proteome profiles

**Figure 3:**
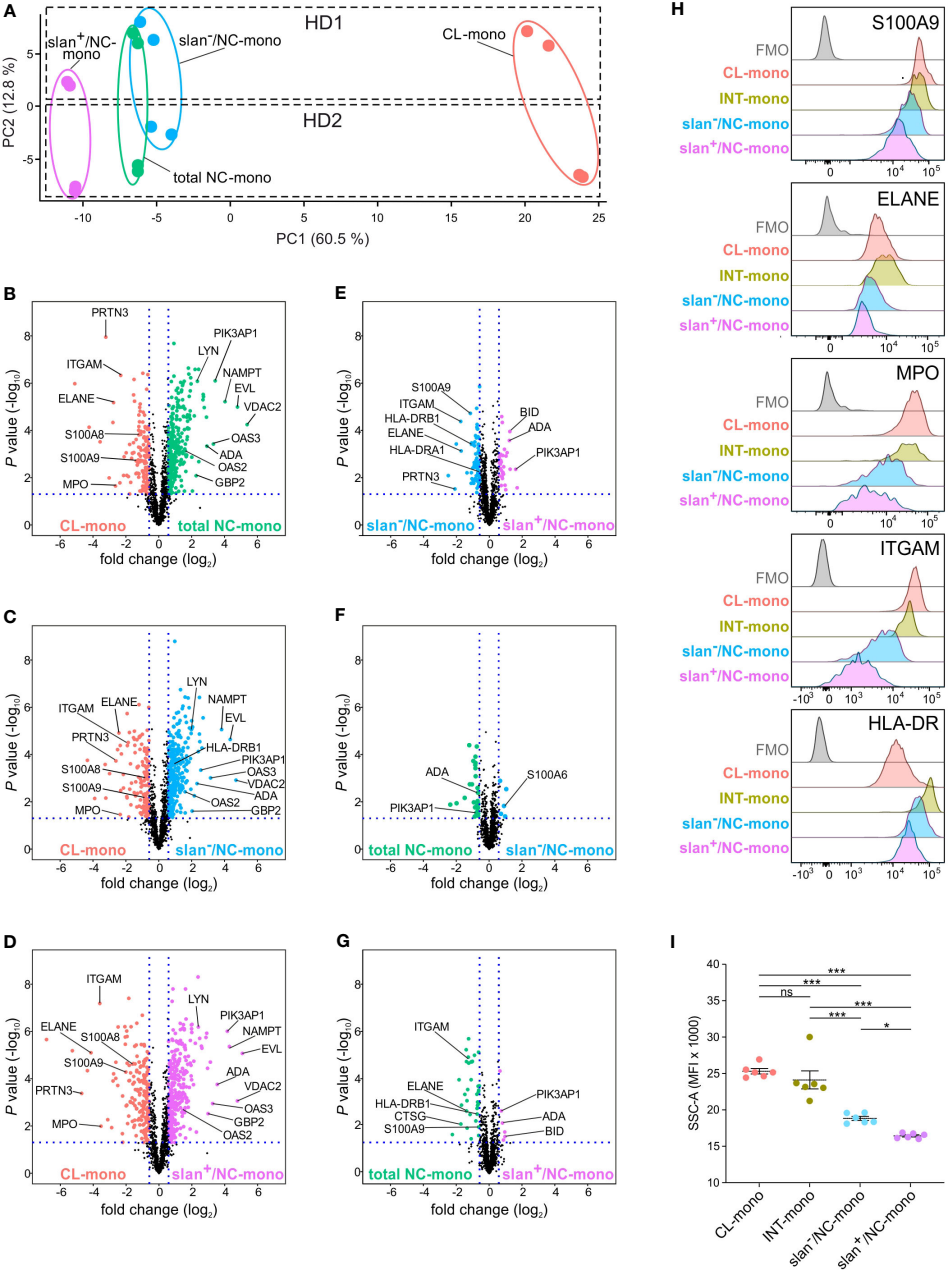
Proteomic profiles of CL-, total NC-, slan^−^/NC- and slan^+^/NC-monocytes. **(A)** PCA of the LFQ protein intensities obtained from CL- (salmon dots), total NC- (green dots), slan^+^/NC- (purple dots) and slan^−^/NC- (light blue dots) monocytes sorted from two healthy donors (HD1 and HD2). Two technical duplicates were performed for each donor, and calculations were made using Perseus software. **(B-G)** LFQ-based volcano plots showing the differentially expressed proteins in CL-, INT-, total NC-, slan^−^/NC- and slan^+^/NC-monocytes. The log_2_ fold change difference (x-axis) is plotted against the corresponding -log_10_ of *P* value (y-axis). Six pair-wise comparisons of proteomes were made: CL- *vs* NC- **(B)**, CL- *vs* slan^−^/NC- **(C)**, CL- *vs* slan^+^/NC- **(D)**, slan^−^/NC- *vs* slan^+^/NC- **(E)** NC- *vs* slan^−^/NC- **(F)** and NC- *vs* slan^+^/NC- **(G)** monocytes. Every dot represents a protein. Differentially expressed proteins (fold change > 1.5, FDR < 0.05 and S0 > 0.1) in CL-, total NC-, slan^+^/NC- and slan^−^/NC-monocytes are represented in salmon, green, light blue and purple dots, respectively. **(H)** CL-, INT-, slan^−^/NC- and slan^+^/NC-monocytes were identified by flow cytometry, using CD14, CD16 and slan membrane expression among Lin^−^ HLA-DR^+^ cells within permeabilized PBMCs. Histograms show the staining of intracellular or membrane expression of S100A9, ELANE, MPO, ITGAM (CD11b) and HLA-DR in monocyte subgroups. FMO (Fluorescence Minus One) control for total PBMC is also displayed. A representative experiment out of 3 is shown. **(I)** Side scatter (SSC-A) median fluorescence intensity (MFI) of CL-, INT-, slan^−^/NC- and slan^+^/NC-monocytes as identified by flow cytometry within PBMCs. Dots show the median of the MFI obtained in every independent experiment (n=6). Means ± SEM are reported. Asterisks indicate significant differences (*P < 0.05, ***P < 0.001 by one-way ANOVA followed by Tukey’s post test).

Then, label-free nLC-MS/MS experiments were performed to investigate if, and how much, slan^+^/NC- and slan^−^/NC-monocytes differ at the proteomic level, either between themselves, or from total NC-monocytes. Whole-cell extracts of total NC-, slan^+^/NC- and slan^−^/NC-monocytes from two different donors were enzymatically digested and analyzed in duplicate by nano LC- coupled with Orbitrap Q-exactive plus analyser. Quantitative analysis performed by a combination of MaxQuant and Perseus software identified a total of 1377 proteins across all samples based on label-free quantification [LFQ] values (sample t test, *P* value < 0.05). As shown in **Figure 3A**, the NC/slan^−^/slan^+^-monocyte group was clearly separated from CL-monocytes by PCA, along PC1 component. Samples of the NC/slan^−^/slan^+^-monocyte group were found close to each other, indicating less pronounced differences among them. However, while slan^+^/NC-monocyte proteome partially differed from the total NC- and slan^−^/NC-monocyte proteomes, the latter two substantially overlapped (**Figure 3A**). Moreover, consistent with bulk RNA-seq results (**Figure 2A**), slan^+^/NC-monocytes consisted of the cell population more distant from CL-monocytes than total NC- and slan^−^/NC-monocytes by PC1 (**Figure 3A**). On the other hand, PC2 was found to clearly separate the biological replicates (HD1 from HD2) (**Figure 3A**).

Next, the differences in proteome expression between CL monocytes and each of the total NC-, slan^−^/NC- and slan^+^/NC-monocyte populations, as well as among total NC-, slan^−^/NC- and slan^+^/NC-monocytes, were assessed by pairwise comparison analysis (*P* value ≤ 0.05 and fold-change ≥ 1.5). As shown by volcano plots, 138, 132 and 197 proteins were more significantly expressed in CL-monocytes than in, respectively, total NC-, slan^−^/NC- and slan^+^/NC-monocytes (**Figures 3B–D**; **Supplementary Table 1**). Conversely, total NC-, slan^−^/NC- and slan^+^/NC-monocytes were found to express higher levels of 296, 300 and 314 proteins, respectively, than CL-monocytes (**Figures 3B–D**; **Supplementary Table 1**). Of note, in addition to the expected ITGAM (CD11b), S100A9 and S100A8 (8, 22), proteases typically associated with azurophilic granules (AG) of neutrophils, including AZU1, PRTN3, ELANE and MPO, were identified among the proteins more expressed in CL-monocytes than in total NC-, slan^−^/NC- and slan^+^/NC-monocytes. Among the proteins more expressed in total NC-, slan^−^/NC- and slan^+^/NC-monocytes than CL-monocytes (**Figures 3B–D**; **Supplementary Table 1**), some (such as PIK3AP1, EVL and LYN) were expected based on the transcriptomic signature of NC-monocytes (8, 22), while others, including VDAC2 and NAMPT, were not. Interestingly, an enrichment of proteins encoded by interferon stimulated genes (ISGs, such as ADA, OAS1, OAS2, OAS3, GBP2, IFI16 and IFI30) was also observed in total NC-, slan^−^/NC- and slan^+^/NC-monocytes (**Figures 3B–D**; **Supplementary Table 1**).

Remarkable differences at the proteomic level were observed also between slan^−^/NC- and slan^+^/NC-monocytes, with 67 and 31 proteins found as significantly more expressed in, respectively, slan^−^/NC- and slan^+^/NC-monocytes, than in the related counterparts (**Figure 3E**; **Supplementary Table 1**). Interestingly, some of the proteins found more expressed in slan^−^/NC-monocytes [such as S100A9, ITGAM, ELANE, CTSG and PRTN3 (**Table 1**)] are characteristic of CL-monocytes (**Figure 3B**; **Supplementary Table 1**), in agreement with the GSEA analysis of the RNA-seq data (**Figure 2D**). These results reflect the lower distance between slan^−^/NC-monocytes and CL-monocytes seen in PCA (**Figure 3A**). In addition, we also observed higher expression of HLA-DR proteins in slan^−^/NC-monocytes than in CL- (**Figure 3C**; **Supplementary Table 1**) or slan^+^/NC-monocytes (**Figure 3E**; **Supplementary Table 1**), suggesting a closer similarity between slan^−^- and INT-monocytes. Moreover, by comparing slan^−^/NC- with slan^+^/NC- monocytes (**Figure 3E**; **Table 1**), we observed that the latter cells display an increased expression of NC proteins shown in **Figure 3B**, such as BID, PIK3AP1 and ADA.

**Table 1:** Most relevant differentially expressed proteins between slan^+^/NC- and slan^−^/NC-monocytes (*P* value ≤ 0.05 and fold-change ≥ 2).

proteins more expressed in slan^+^/NC-monocytes		
Symbol	Name	fold change
SNRPE	Small nuclear ribonucleoprotein E	3,24
PIK3AP1	Phosphoinositide 3-kinase adapter protein 1	3,04
RAB8A	Ras-related protein Rab-8A	2,40
BID	BH3-interacting domain death agonist	2,37
ADA	Adenosine deaminase	2,32
ARF5	ADP-ribosylation factor 5	2,12
IGHG1	Immunoglobulin heavy constant gamma 1	2,02
PICALM	Phosphatidylinositol-binding clathrin assembly protein	2,00
proteins more expressed in slan^−^/NC-monocytes		
Symbol	Name	fold change
CTSG	Cathepsin G	5,64
PRTN3	Myeloblastin	4,27
NUMA1	Nuclear mitotic apparatus protein 1	4,02
ITGAM	Integrin alpha-M	3,34
ELANE	Neutrophil elastase	3,28
LYZ	Lysozyme	2,56
S100A9	Protein S100-A9	2,23
CPVL	Probable serine carboxypeptidase CPVL	2,15
TARS	Threonine–tRNA ligase, cytoplasmic	2,13
HLA-DRB1	HLA class II histocompatibility antigen, DRB1 beta chain	2,13
HINT1	Histidine triad nucleotide-binding protein 1	2,11
GGH	Gamma-glutamyl hydrolase	2,08
TSPO	Translocator protein	2,01

Subsequent pairwise comparison analysis revealed that total NC-monocytes display 40 differentially expressed proteins with slan^−^/NC- monocytes (**Figure 3F**; **Supplementary Table 1**) and 46 with slan^+^/NC-monocytes (**Figure 3G**; **Supplementary Table 1**). Importantly, even when compared with total NC-monocytes, slan^+^/NC-monocytes exhibited higher expression of NC-monocyte proteins (such as BID, ADA and PIK3AP1) and lower expression of CL-monocytes proteins (such as S100A9, ITGAM and ELANE) (**Figure 3G**). To validate proteome results, we performed intracellular and membrane flow cytometry analysis of discrete proteins, however using total PBMCs. As shown in **Figure 3H**, we could confirm that CD11b, HLA-DR, S100A9, ELANE and MPO are more strongly expressed in INT- and CL- than in NC-monocytes. In addition, the expression of these proteins resulted significantly higher in slan^−^/NC- than in slan^+^/NC-monocytes (**Figure 3H**), confirming proteomic results (**Figure 3G**). We also observed that the SSC parameter (measuring the granularity levels) of slan^+^/NC-monocytes results lower than the SSC parameter displayed by the CL-, INT-, slan^−^/NC- and even total NC-monocytes (**Figure 3I**), consistent with the differential expression of AG proteins in slan^+^/NC- versus slan^−^/NC-monocytes (**Figure 3G**). In sum, the data shown above suggest that, at the proteomic level, slan^+^/NC- and slan^−^/NC-monocytes differ from each other, as well as that slan^+^/NC-monocytes express higher levels of NC-monocytes specific proteins.

### Heterogeneity of monocytes by single-cell RNA sequencing

**Figure 4:**
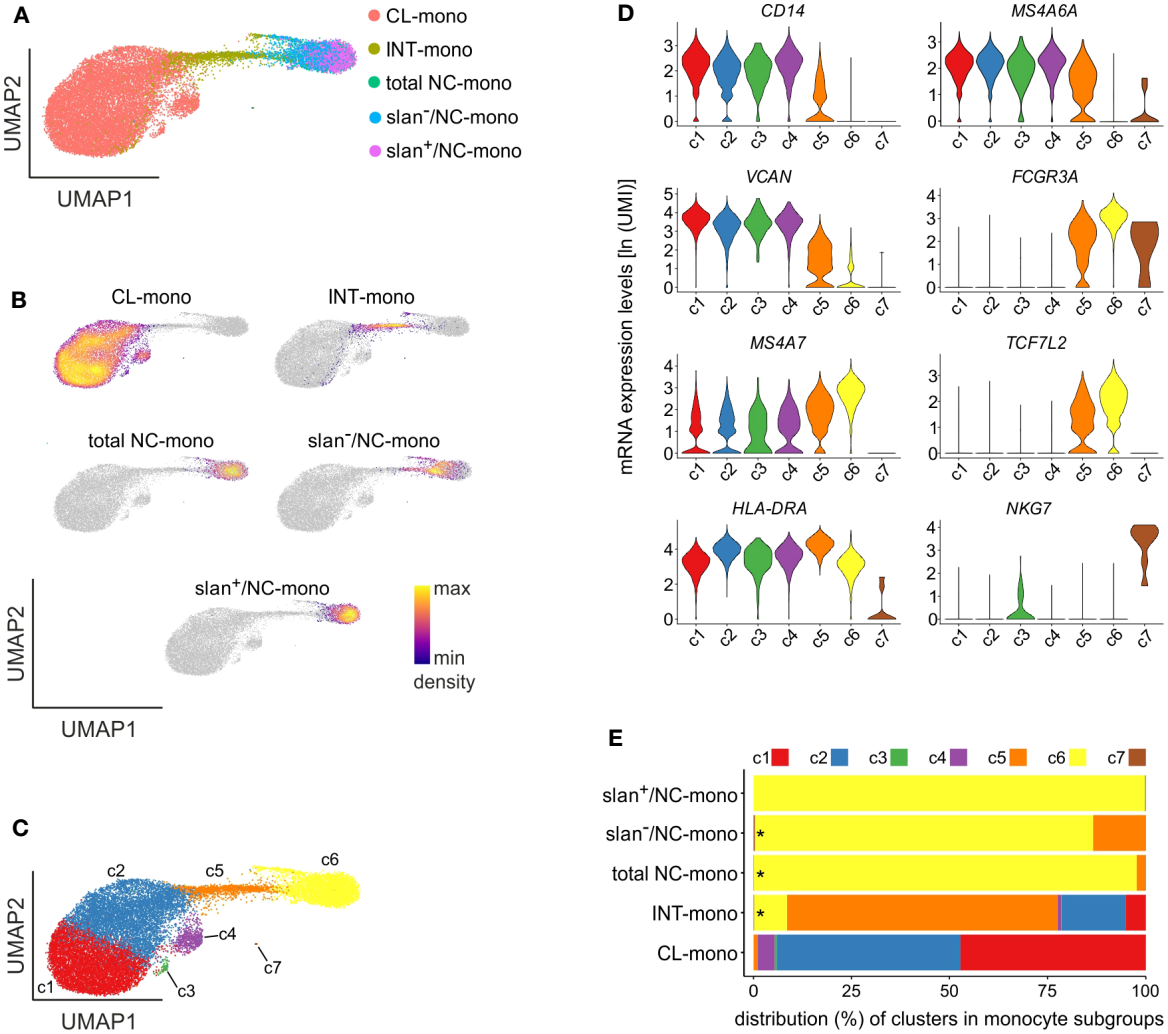
scRNA-seq experiments of monocyte subgroups. **(A)** UMAP of scRNA-seq profiles of CL- (salmon), INT- (yellow ochre), total NC- (green), slan^−^/NC- (light blue) and slan^+^/NC- (purple) monocytes sorted from the blood of two different HDs. **(B)** Density plots of CL-, INT-, total NC-, slan^−^/NC- and slan^+^/NC-monocytes overlaid on the UMAP of panel **(A)** Density of cells in each plot refers to the indications of the colored bar. **(C)** UMAP plot showing 7 clusters (c1-c7) determined by Louvain clustering analysis. **(D)** Violin plots showing the mRNA expression levels [as ln(UMI)] of genes specific of CL- (*CD14*, *MS4A6A* and *VCAN*), NC- (*FCGR3A*, *MS4A7* and *TCF7L2*) and INT- (*HLA-DRA*) monocytes, as well as NK cells (*NKG7*), across c1-c7 cells. **(E)** Stacked bar graph shows the relative abundances of c1-c7 cells (indicated as percentages) in CL-, INT-, total/NC-, slan^+^/NC- and slan^−^/NC-monocytes. Asterisks indicate the presence of c7 cells. Cluster colors used in panels **D**, **E** are the same of those shown in panel **C**.

Data shown in **Figures 2**, **3** do not exclude that slan^−^/NC-monocytes could have been contaminated by few CL- or INT-monocytes, caused by unavoidable imperfect sorting procedures. Therefore, total NC-, slan^−^/NC- and slan^+^/NC-monocytes from another group of HDs were sorted along with INT- and CL-monocytes (for comparison purposes), and then subjected to single-cell RNA sequencing (scRNA-seq), since it currently represents the most precise method to unequivocally identify all the various cell populations within heterogeneous cell samples. Totally, we sequenced 20890 cells, which decreased to 19085 after quality control. Dimensionality reduction by UMAP (26) clearly demonstrated a net segregation of the NC/slan^+^/slan^−^- and CL-monocyte groups, with INT-monocytes placing exactly in between them (**Figures 4A, B**), as expected (8, 12, 27, 28). Moreover, in line with the bulk transcriptomic (**Figure 2A**) and proteomic (**Figure 3A**) results, the same UMAP confirmed that slan^−^/NC-monocytes stand closer to CL-and INT-monocytes than to slan^+^/NC-monocytes (**Figures 4A, B**).

By performing unbiased, graph-based, clustering by Seurat (29), we could identify 7 discrete cell clusters (**Figure 4C**), 4 of which unequivocally attributable to CL-monocytes (c1-c4), 1 to INT-monocytes (c5) and 1 to NC-monocytes (c6), based on the expression of specific genes characterizing the three main monocyte subgroups. In fact, c1-c4 and c6 cells were found to primarily express genes typical of, respectively, CL-monocytes (such as *CD14*, *MS4A6A* and *VCAN*) and NC-monocytes (such as *FCGR3A*, *MS4A7* and *TCF7L2*) (8) (**Figure 4D**), while c5 cells were found to express high levels of HLA-DR genes (**Figure 4D**), which is a distinctive feature of INT-monocytes (30). A cluster consisting of 8 cells (c7) (**Figure 4C**), expressing typical NK genes such as *FCGR3A* and *NKG7*, was attributable to contaminating NK cells (**Figure 4D**). These latter cells were included in total NC-, slan^−^/NC-, INT- and CL-monocytes, but not slan^+^/NC-monocytes (**Figure 4E**; **Supplementary Figure 2A**), corroborating our bulk RNA-seq experiments (**Figure 2C**). Distribution of scRNA-seq cell clusters within the monocyte subgroups highlighted that the NC/slan^+^/slan^−^-monocyte group include the great majority of c6 cells, which were found to also contain a few INT-monocytes, but no CL-monocytes at all (**Figure 4E**). Interestingly, while slan^+^/NC-monocytes consisted exclusively of c6 cells (by 99.73%), slan^−^/NC- and total NC-monocytes were found to also include c5 cells (by 13.38% and 2.22%, respectively) (**Figure 4E**). Finally, while INT-monocytes were found to consist mostly of c5 cells (by 68.9%), CL-monocytes included mainly c1 (by 47.23%) and c2 (by 46.88%) (**Figure 4E**).

**Figure 5:**
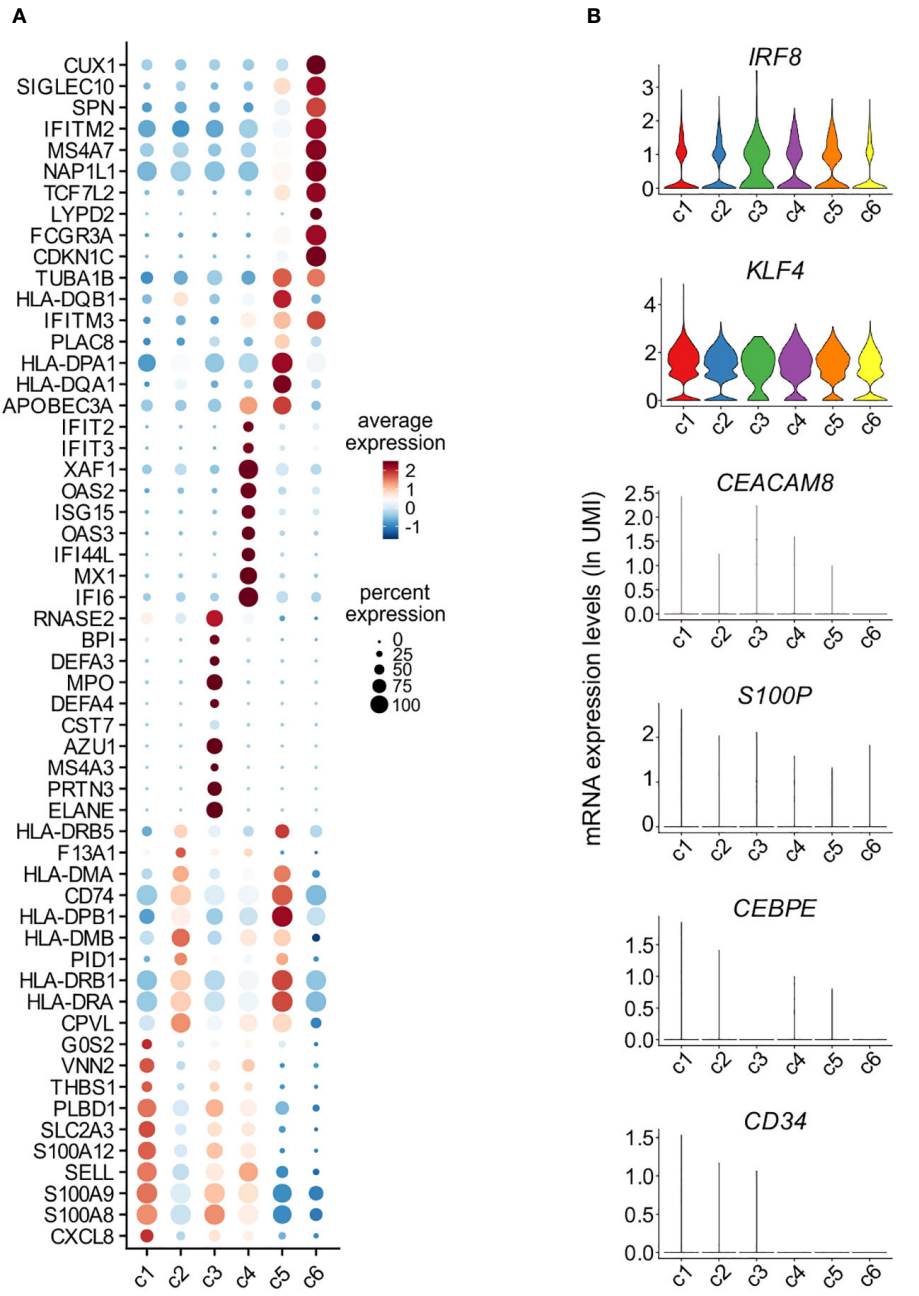
Characterization of monocyte clusters identified by scRNA-seq analysis. **(A)** Dot plot showing the top 10 genes, sorted by average log fold change, specific for the monocyte clusters (c1-c6) as identified and shown in **Figure 4C**. Dot size represents the percentage of cells in each cluster with more than one read of the corresponding gene. The color intensity of the dots represents the level of expression of the genes. **(B)** Violin plots showing the mRNA expression levels (as ln(UMI)) of selected genes (i.e., *IRF8*, *KLF4, CEACAM8, S100P, CEBPE* and *CD34*) across c1-c6 cells.

Assessment of the DEGs characterizing c1-c6 monocytes resulted in the identification of 990 genes, of which the most representative are indicated in **Figure 5A**. Precisely: c1 cells were found to express high levels of genes typical of CL-monocytes (such as *S100A8*, *S100A9*, *S100A12* and *THBS1*) (8) and other typical of mature neutrophils (such as *CXCL8*, *SELL* and *G0S2*) (31)(**Figure 5A**); c2 was found to include cells expressing MHC class II genes (such as *HLA-DRA, HLA-DRB1, HLA-DPB1, HLA-DQA1, HLA-DQB1* and *HLA-DPA1*), however at lower levels than those observed in c5 (the cluster representing INT-monocytes); c4 cells, instead, were found to highly express ISGs such as *IFI6*, *ISG15* and *MX1*, while c3, consisting of only 73 cells, was characterized by the expression of genes encoding for AG proteins of neutrophils (i.e., *MPO*, *ELANE* and *AZU1*) and defensins (*DEFA3*, *DEFA4*) (**Figure 5A**), typically present in neutrophil and monocyte precursors (32). The fact that c1 and c3 cells were found to also express typical monocyte genes, such as *IRF8* and *KLF4*, but not neutrophil-specific genes, such as *CEACAM8*, *S100P* and *CEBPE* (**Figure 5B**), excludes their contamination with mature neutrophils or neutrophil precursors. In addition, we could exclude that c3 cells could correspond to circulating cMoPs for the absence of transcripts commonly associated to immature cells (such as *CD34*) (**Figure 5B**). c6 cells were found to express very high levels of *FCGR3A, MS4A7, IFITM2* and *TCF7L2* mRNAs belonging to the NC-signature genes (**Figure 5A**) (8), and was thus better characterized in a separate analysis, as detailed in the next paragraph. Altogether, these experiments indicate that differently from slan^−^/NC- or total NC-monocytes, the slan^+^/NC-monocytes exclusively consist of cells from the c6 cell cluster, which represent the prototypical NC monocytes.

### scRNA-seq reveals distinct cluster distribution and gene expression patterns in total NC-, slan^+^/NC and slan^−^/NC-monocytes

**Figure 6:**
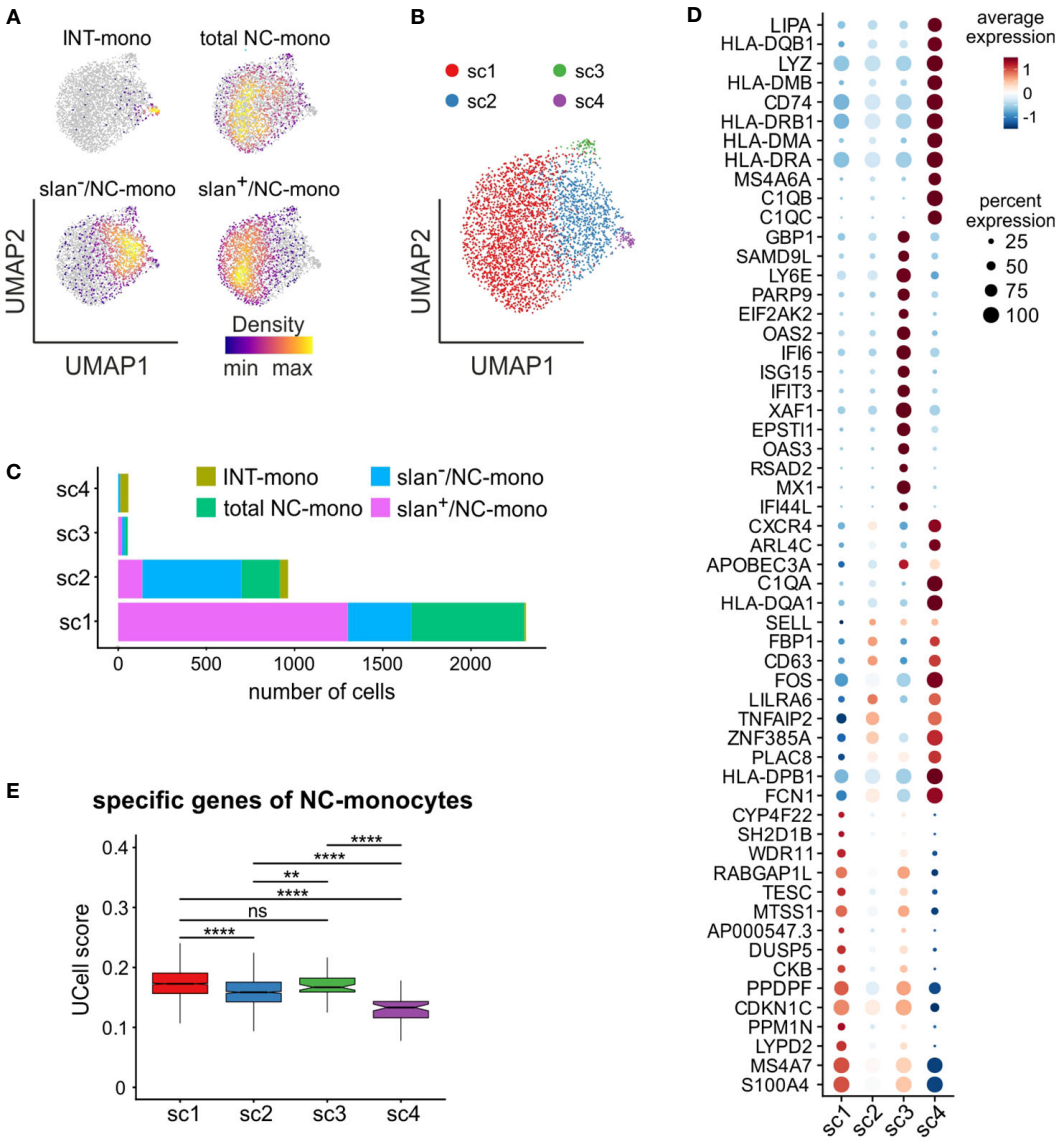
**In-depth analysis of the non-classical monocyte cluster 6.** scRNA-seq profiles of cells from c6 of **Figure 4C** were retrieved and subjected to further analysis. **(A)** Density plots of INT-, total/NC-, slan^−^/NC- and slan^+^/NC-monocytes overlaid on the UMAP plot of c6. Density of cells in each plot is depicted according to the indications of the colored bar. **(B)** UMAP plot showing scRNA-seq profiles of c6 non-classical monocytes grouped in 4 sub-clusters (sc1-sc4), identified by clustering analysis. **(C)** Stacked bar graph showing the number of INT-, total/NC-, slan^+^/NC- and slan^−^/NC- in sc1-sc4. **(D)** Dot plot showing the top 15 marker genes, sorted by average log fold change, associated with the non-classical monocyte sub-clusters identified in panel **(B)** Colors of the dots indicate the average expression of each gene in each sub-cluster scaled across all clusters. Dot size represents the percentage of cells in each sub-cluster with more than one read of the corresponding gene. **(E)** Box plot showing the UCell score of non-classical specific genes retrieved from reference (22) in sc1-sc4. The box plot shows the median with the lower and upper quartiles representing a 25th to 75th percentile range and whiskers extending to 1.5 × interquartile range (IQR). ns, not significant, **P < 0.01, ****P < 0.0001 by unpaired two-samples Wilcoxon test using the Bonferroni method correction for multiple comparisons.

Next, we performed a separate bioinformatic analysis of c6, to directly compare total NC-, slan^+^/NC- and slan^−^/NC-monocytes in the absence of cells from c5 (INT-monocytes) and c7 (NK cells). As shown by **Figure 6A**, slan^+^/NC- and slan^−^/NC-monocytes occupy distinct regions in the UMAP, while total NC-monocytes are more broadly distributed. In addition, those few INT-monocytes present in c6 (**Figure 4E**) reside in a small peripheral region of the UMAP (**Figure 6A**). To better identify the differences between slan^+^/NC- and slan^−^/NC-monocytes at the transcriptomic levels, we performed a re-clustering analysis of c6, and obtained 4 new sub-clusters (that we named sc1/sc4) (**Figure 6B**). sc1 and sc2 represented the largest sub-clusters, sc1 being enriched in slan^+^/NC-monocytes (for more than 76.92%), as opposed to sc2, enriched in slan^−^/NC-monocytes (**Figure 6C**). By contrast, both sc3 and sc4 consisted of very few cells, sc4 mainly including INT-monocytes, while sc3 equally consisting of total NC-, slan^+^/NC- and slan^−^/NC-monocytes (**Figure 6C**; **Supplementary Figure 2B**). Interestingly, sc1 cells were found to express high levels of genes distinctive of the NC-signature (such as *LYPD2*, *PPM1N*, *CKB*, *MTSS1*, *CYP4F22* and *TESC*), while sc2 cells, similarly to sc4, were found to also express genes typical of INT-monocytes (such as *PLAC8* and *CD63*) (**Figure 6D**). In addition, sc4 cells were found to express high levels of HLA-DR genes (**Figure 6D**), in accordance with the prevalence of INT-monocytes in this cluster (**Figure 6C**), as well as of C1Q genes (**Figure 6D**). Actually, *C1QA*, *C1QB*, and *C1QC* gene expression was also analyzed in the UMAP including all monocyte subgroups (**Figure 4A**). We found that *C1QA*, *C1QB*, and *C1QC* genes were mainly expressed by a restricted group of cells localized in a confined region of the NC-monocyte cluster (**Supplementary Figure 2C**). Consistently, sc4 monocytes were found to be co-located with monocytes exhibiting high C1Q expression. (**Supplementary Figure 2D**). Differently from sc4, sc3 cells were found to highly express ISGs (**Figure 6D**), therefore representing the NC-monocyte counterpart of the CL-monocytes present in c4 (**Figure 5A**). Finally, confirming previous data from bulk RNA-seq analysis (**Figure 2D**), we found that sc1, mainly composed of slan^+^/NC-monocytes, represented the sub-cluster accumulating higher levels of the NC-monocyte signature genes, as evidenced by the box plot of **Figure 6E**.

## Discussion

While it is well established that the slan antigen marks NC-monocytes (2), it remains to be clarified whether both slan^+^/NC- and slan^−^/NC- effectively represent prototypical NC-monocytes. If the slan^+^/NC-monocytes, and not the slan^−^/NC-monocytes, are characterized by NC-monocyte features, the slan marker could be reasonably utilized to more correctly identify and isolate NC-monocytes (2). In fact, given its greater stability and reliability than CD14 or CD16 under stimulatory and/or pathological conditions (4) - as also shown here for CD16 -, slan marker usage would eliminate eventual flaws caused by monocyte staining with anti-CD16 and/or anti-CD14 (2).

In this study, we investigated if, and if so, how much, slan^+^/NC- and slan^−^/NC-monocytes differ at the transcriptomic and proteomic level by multi-omics approaches consisting of bulk RNA-seq, scRNA-seq and MS-based proteomics. In fact, while several studies have focused on the transcriptomes of total monocytes (7, 9, 11, 28, 33, 34), specific comparisons among total NC-, slan^+^/NC- and slan^−^/NC-monocyte transcriptomes have been performed in few reports only (11, 12). For instance, Cros and colleagues (12) found that total NC-, slan^+^/NC-, slan^−^/NC-monocytes exhibit no transcriptomic differences, as evidenced by PCA and hierarchical clustering analysis. Using the same computational methods, our data aligned with the aforementioned study (12). However, authors did not utilize differential expression analysis or GSEA, which instead we employed here to identify specific differences between slan^+^/NC- and slan^−^/NC-monocytes. In a more recent study, Hamers et al. (11) identified one group of slan^−^/NC-monocytes and two of slan^+^/NC-monocytes by CyTOF analysis of blood monocytes. In particular, slan^+^/NC-monocytes were distinguished, among themselves, by their positive or negative CD9 membrane expression. However, it was then uncovered that the division CD9^+^ and CD9^−^ slan^+^-monocytes was not correlated to a genuine differential CD9 expression (11). In fact, CD9 was found expressed by platelets bound to monocytes, rather than monocytes themselves (11). Consistently, some of the genes reported by Hamers et al. (11) to be upregulated in CD9^+^slan^+^-monocytes, such as *MMRN1*, *SELP*, *ITGA2B*, and *TUBB1*, are known to be primarily expressed in platelets and megakaryocytes (35). In our bulk RNA-seq, as well as scRNA-seq, no expression of *MMRN1*, *SELP*, *ITGA2B*, and *TUBB1* was detected, hence ruling out the presence of platelet-monocyte aggregates in our samples. By comparing the gene expression profiles of slan^+^/NC- and slan^−^/NC-monocytes, Hamers et al. identified only 20 DEGs, indication that these two subgroups were quite similar at the transcriptomic level (11). Likewise, only 29 genes resulted differentially expressed between slan^+^/NC- versus slan^−^/NC-monocytes in our bulk RNA-seq studies, even though slan^+^/NC-monocytes were found to display, by GSEA, an enrichment of genes belonging to a NC-monocyte signature. GSEA is in fact a very potent method to determine whether a group of functionally related set of genes (such as the NC-monocyte signature) shows statistically significant differences among different or similar cell types (i.e. slan^+^/NC- versus slan^−^/NC-monocytes) (25). However, according to our datasets, none of the 20 DEGs reported by Hamers et al. (11) were found to be differentially expressed by slan^+^-/NC- versus slan^−^/NC-monocytes. In such regard, it should be pointed out that, compared to Hamers et al. (11), we used a different gating strategy to isolate monocyte subgroups, as well as a more stringent cut-off (> 1 FPKM) to define whether a given gene was expressed or not in our samples. Hofer et al. (7) too performed transcriptomic analysis of slan^+^/NC-monocytes, ultimately proposing to classify CD16^+^slan^−^- monocytes within INT monocytes, which would therefore include both CD14^dim^ and CD14^++^-monocytes. Our study however indicates that most of the CD16^+^slan^−^-monocytes are included in c6 of our scRNA-seq clusters, corresponding to NC-monocytes, and only a 13.4% of slan^−^-monocytes are included in c5, the cluster representing INT-monocytes. It should be pointed out that, in Hofer et al.’s study (7), the transcriptome of slan^+^/NC-monocytes was compared to that of total CD14^dim/++^CD16^+^slan^−^-monocytes that also include INT-monocytes. Therefore, such an approach excludes possible comparisons with our results. In any case, our bulk RNA-seq, and even scRNA-seq analysis, clearly distinguished slan^−^/NC- from INT-monocytes, thus excluding that they consist of a unique population.

Our scRNA-seq experiments confirmed previous information demonstrating that human monocytes are actually more heterogeneous than the three subgroups identifiable by evaluating CD14 and CD16 membrane expression (27, 36–38). In our hands, human monocytes were found to consist of 6 clusters, four of them representing CL-monocytes (i.e., c1-c4), one representing INT-monocytes (i.e., c5) and one NC-monocytes (i.e., c6). Of the CL-monocyte clusters, c1 and c3 cells recalled the neutrophil-like monocytes originally identified in mice (39), and then found in humans too (27, 36, 38, 40). In fact, both c1 and c3 cells were found to display high levels of *S100A8*, *S100A9*, *CXCL8*, *SELL* and *VEN2* mRNAs, which are all genes characteristic of neutrophils. In addition, c3 cells were found to also express genes encoding for AG proteins, that are typically expressed by myeloid precursors (32). Whether c3 cells correspond to a novel circulating neutrophil-like monocyte precursor, certainly more mature than the cMoPs, should be further explored. With regard to c4, we detected the presence of ISG-expressing cells, in line with recent findings by Rigamonti et al. (27). On the other hand, c2 cells were found to display a high expression of HLA-DR encoding genes, although comparatively lower than that by c5 cells representing INT-monocytes. It should be remarked that in other scRNA-seq studies were identified a lower (n= 4-5) (36–38) or a higher (n= 8) (27), number of monocyte clusters than we did here. Such a variability is likely determined by different numbers of cells analyzed in the various studies, and/or by different parameters used in performing clustering analysis.

Recently, also scRNA-seq studies of slan^+^/NC- and slan^−^/NC-monocytes have pointed out that slan expression does not discriminate different cell subgroups at the transcriptomic level (41). Our study substantially confirms this view, but also reveals that slan^+^/NC- and slan^−^/NC-monocytes, even if very similar, display some peculiar differences. In fact, a not negligible fraction (approximately 13.4%) of slan^−^/NC-monocytes were found in c5, that is the cluster corresponding to the INT monocytes. Moreover, when performing a separate analysis of c6, we observed that slan^+^/NC-monocytes represent a more homogeneous cell population than the slan^−^/NC-monocytes within it. In fact, more than 87% slan^+^/NC-monocytes were found included in a single subcluster (sc1), by the way displaying the highest expression of specific NC-monocyte genes. Interestingly, within c6, we also identified a subcluster (sc4), composed of INT- and slan^−^/NC-monocytes (but not slan^+^/NC-monocytes), which included cells expressing high levels of C1Q genes, i.e., molecules important for the activation of the classical complement cascade and efferocytosis (42). Cell clusters of circulating monocytes highly expressing C1Qs genes have been identified also in other scRNA-seq studies (27, 43–45), which altogether confirm bulk RNA-seq experiments evidencing an elevated expression of C1Qs genes by NC- and INT-monocytes (23, 28, 46). Whether sc4 cells have specific biological function has yet to be investigated.

By performing proteomic experiments, we identified more than 1000 differentially expressed proteins among CL-, total NC-, slan^+^/NC- and slan^−^/NC-monocytes. It should be mentioned that previous studies have performed proteomic analysis of slan^+^/NC- and slan^−^/NC-monocytes by mass cytometry (10, 11), but only at the level of membrane proteins. Other studies instead compared the proteomes of monocyte subgroups without discriminating slan^+^/NC- from slan^−^/NC-monocytes (47, 48). The study by Segura et al. (48), for instance, compared CL-monocytes to NC-monocytes (these last including also a 30% of INT-monocytes), which makes impossible to compare their results with ours. Instead, many of the differentially expressed proteins in CL- and NC- monocytes identified by Zhao et al. (47), even if in a lower number, were recognized as differentially expressed also in our datasets. Our results clearly indicate that slan^−^/NC-monocytes, compared to slan^+^/NC-monocytes, express higher levels of proteins present in CL-monocytes (such as S100A9, CD11b and AG proteins) (47) and/or in INT-monocytes (such as HLA-DR). By contrast, slan^+^/NC-monocytes were found to express higher levels of proteins expressed by NC subgroup (such as LYN, BID and PIK3AP1) (47) than slan^−^/NC-monocytes do. These results not only further support the data from bulk RNA-seq and scRNA-seq analysis, but also suggest that protein analysis better discriminate slan^+^/NC- from slan^−^/NC-monocytes than mRNA analysis. It is nonetheless important to remark that the single cell transcriptome and proteome analyses in this study are based on a sizable number of cells, but these are from only two different blood donors each. To reach a more consistent statistical power many more donors should have been analyzed by these two “omics” methodologies. We recognize that this might be a potential limitation of our study because the genetic and cellular characteristics of individuals vary widely within the human population and, therefore, we are aware that our data may not cover the entire heterogeneity of humankind. However, the fact that four different methods (i.e., bulk RNAseq, scRNAseq, proteomics and flow cytometry), combined with the utilization of diverse donors for each of them, have given a similar output, reassure us about the validity of the findings herein presented.

In conclusion, by an accurate characterization of their transcriptomes and proteomes, we provide evidence that slan^+^/NC-monocytes can be defined as prototypical NC-monocytes. Therefore, we substantiate the notion that isolation and identification of NC-monocytes *via* the slan marker should be preferred with respect to the CD14/CD16 marker combination, in order to more precisely study NC-monocytes in health and disease (2).

## Data availability statement

Our data are now publicly available at the Gene Expression Omnibus database (https://www.ncbi.nlm.nih.gov/geo/) under the accession numbers GSE136107 and GSE242314.

## Ethics statement

The studies involving humans were approved by Ethics Committee of the Azienda Ospedaliera Universitaria Integrata di Verona (Italy). The studies were conducted in accordance with the local legislation and institutional requirements. The participants provided their written informed consent to participate in this study.

## Author contributions

NT: Conceptualization, Formal Analysis, Funding acquisition, Investigation, Supervision, Visualization, Writing – original draft, Writing – review & editing. FB-A: Data curation, Formal Analysis, Investigation, Visualization, Writing – original draft. SG: Investigation, Validation, Writing – original draft. AG: Formal Analysis, Investigation, Methodology, Visualization, Writing – original draft, Writing – review & editing, Conceptualization. CM: Formal Analysis, Investigation, Methodology, Writing – original draft. FC: Formal Analysis, Investigation, Writing – original draft. EG: Formal Analysis, Investigation, Visualization, Writing – original draft. IS: Investigation, Writing – original draft. MC: Investigation, Writing – original draft. VB: Conceptualization, Supervision, Writing – original draft. MT: Conceptualization, Supervision, Writing – original draft. MAC: Conceptualization, Data curation, Funding acquisition, Project administration, Supervision, Writing – original draft, Writing – review & editing.
