# Supplementary material for: The slan antigen identifies the prototypical non-classical CD16+-monocytes in human blood
Source: Front Immunol. 2023 Oct 27;14:1287656. doi: 10.3389/fimmu.2023.1287656 (PMC10641684; doi:10.3389/fimmu.2023.1287656)
Supplement: Supplementary file 2 [file Image_1.pdf]

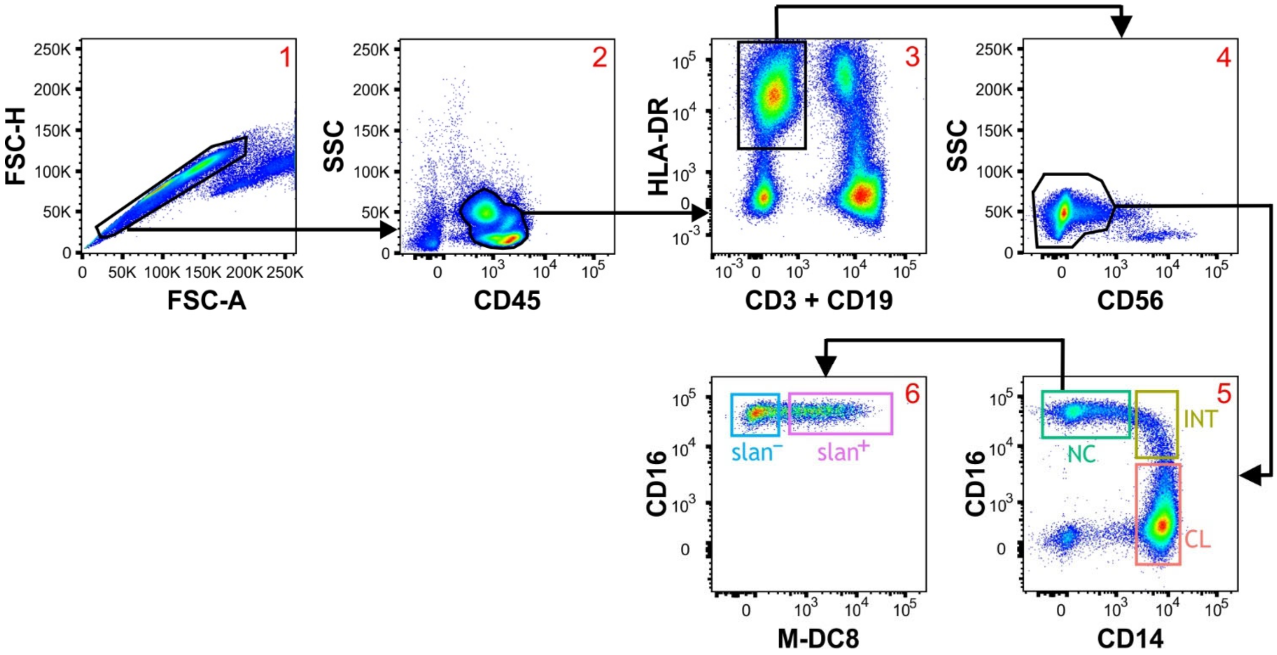

Supplementary Figure 1. Flow cytometry gating strategy to sort blood monocyte subtypes. CL-, INT-, total NC-, slan^{-}/NC- and slan^{+}/NC-monocytes were isolated by cell sorting from human PBMCs by the following gating strategy. At first, we excluded doublets (panel 1), then gated CD45^{+}-cells (panel 2), then gated HLA-DR^{+} lineage CD3/CD19-negative cells (panel 3) and, ultimately, we excluded CD56^{+} NK cells (panel 4). Next, NC-, INT-, and CL-monocytes (respectively evidenced by the green, yellow ochre and salmon gates) were gated using CD14/CD16 markers (Panel 5). Finally, slan^{+}/NC- (purple gate) and slan^{-}/NC- (light blu gate) monocytes were separated within NC-monocytes by MDC8 staining, as shown in panel 6.
