# Supplementary material for: The slan antigen identifies the prototypical non-classical CD16+-monocytes in human blood
Source: Front Immunol. 2023 Oct 27;14:1287656. doi: 10.3389/fimmu.2023.1287656 (PMC10641684; doi:10.3389/fimmu.2023.1287656)
Supplement: Supplementary file 3 [file Image_2.pdf]

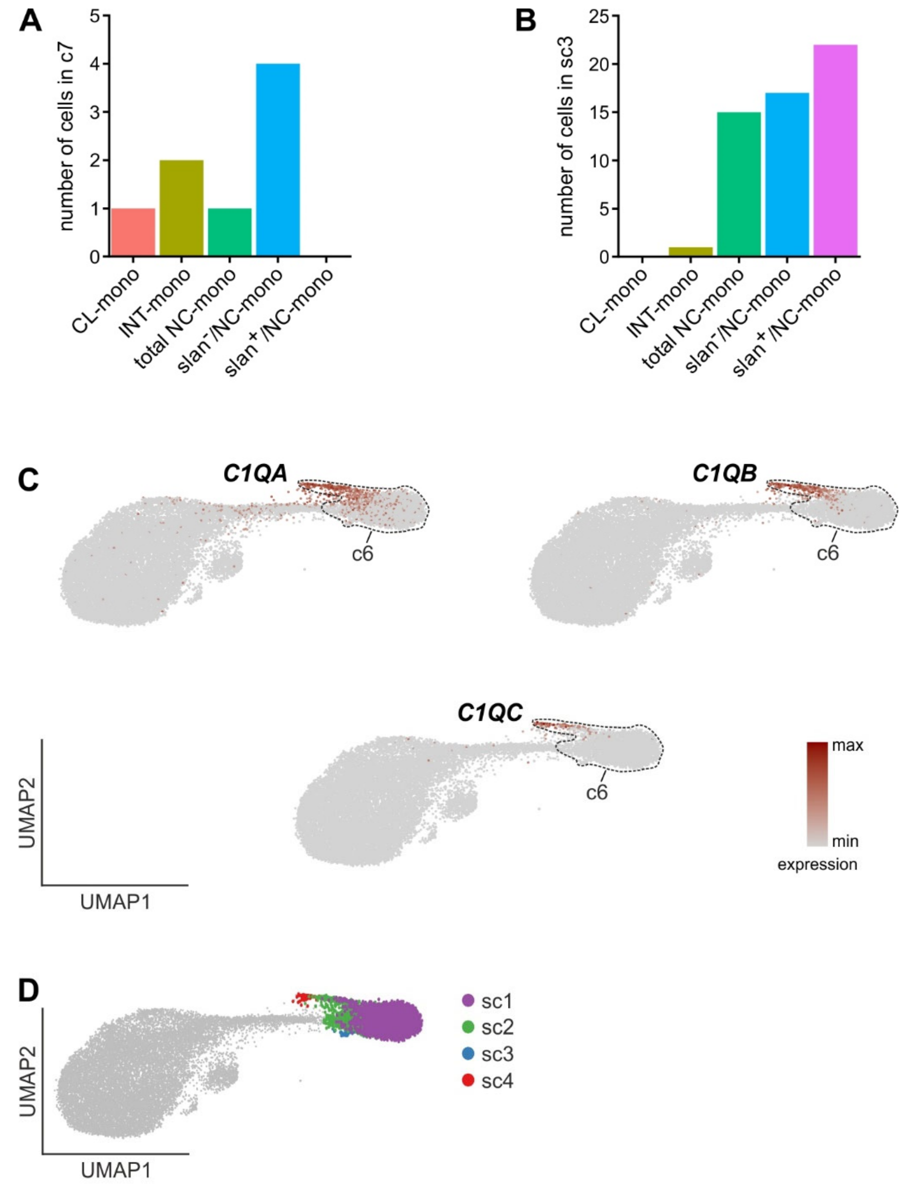

Supplementary Figure 2. Identification of monocyte types in cells of c7, sc3 and among those expressing C1Q genes. Bar graphs indicate the number of CL-, INT-, total NC-, slan^{-}/NC- and slan^{+}/NC-monocytes present in c7 from Figure 4C (panel **A**), and sc3 from Figure 6B (panel **B**). (**C**) Expression patterns of *C1QA*, *C1QB*, and *C1QC* mRNAs projected on the UMAP plot shown in Figure 4. The dotted lines encircle the cells included in c6 (displayed in Figure 4). (**D**) Projection of cells composing sc1-sc4, obtained by the sub-clustering of c6 (Figure 6), into the UMAP plot shown in Figure 4.
